# Light-Induced Infrared Difference Spectroscopy in the Investigation of Light Harvesting Complexes

**DOI:** 10.3390/molecules200712229

**Published:** 2015-07-03

**Authors:** Alberto Mezzetti

**Affiliations:** 1 Laboratoire de Spectrochimie Infrarouge et Raman LASIR—UMR 8516, Université Lille 1, Bat C5, Cité Scientifique, 59655 Villeneuve d’Ascq, France; E-Mail: alberto.mezzetti@libero.it; 2 Institute for Integrative Biology of the Cell (I2BC), Commissariat à l’Energie Atomique et aux Energies Alternatives (CEA), Centre National de la Recherche Scientifique (CNRS), Université Paris-Sud, CEA-Saclay, F-91191 Gif-sur-Yvette, France

**Keywords:** light-harvesting systems, peridinin, LHCII, thylakoids, orange carotenoid protein, step-scan FTIR, infrared difference spectroscopy, photoprotection, rapid-scan FTIR

## Abstract

Light-induced infrared difference spectroscopy (IR-DS) has been used, especially in the last decade, to investigate early photophysics, energy transfer and photoprotection mechanisms in isolated and membrane-bound light harvesting complexes (LHCs). The technique has the definite advantage to give information on how the pigments and the other constituents of the biological system (proteins, membranes, *etc.*) evolve during a given photoreaction. Different static and time-resolved approaches have been used. Compared to the application of IR-DS to photosynthetic Reaction Centers (RCs), however, IR-DS applied to LHCs is still in an almost pioneering age: very often sophisticated techniques (step-scan FTIR, ultrafast IR) or data analysis strategies (global analysis, target analysis, multivariate curve resolution) are needed. In addition, band assignment is usually more complicated than in RCs. The results obtained on the studied systems (chromatophores and RC-LHC supercomplexes from purple bacteria; Peridinin-Chlorophyll-*a*-Proteins from dinoflagellates; isolated LHCII from plants; thylakoids; Orange Carotenoid Protein from cyanobacteria) are summarized. A description of the different IR-DS techniques used is also provided, and the most stimulating perspectives are also described. Especially if used synergically with other biophysical techniques, light-induced IR-DS represents an important tool in the investigation of photophysical/photochemical reactions in LHCs and LHC-containing systems.

## 1. Introduction

Infrared difference spectroscopy (IR-DS) is a biophysical technique that can be particularly helpful in the study of the mechanism of biochemical reactions [1,2]. Differently from other spectroscopic techniques, which bring information essentially on “target” molecules or molecular moieties (chromophoric or fluorescent molecules, paramagnetic species, *etc.*), IR-DS provide information on all—or almost all—the constituent of the system under investigation (protein, membrane, *etc.*), the only exception being the molecules whose vibrations do not give any IR signal (e.g., molecular oxygen, O_2_).

**Figure 1:**
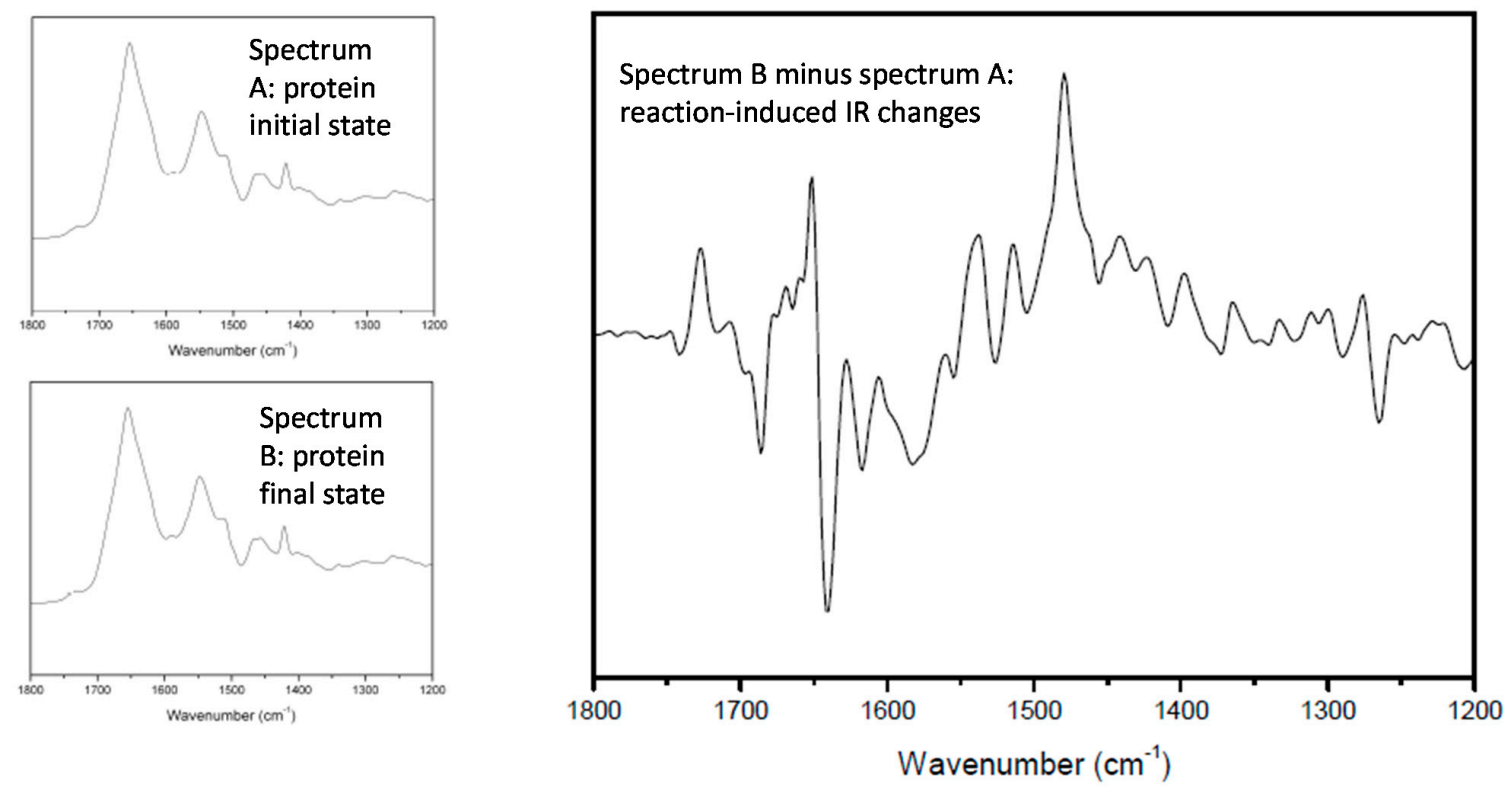
Principle of IR-DS. The figure shows a classical example of photosynthetic reaction, the mono-electronic reduction of the so-called Q_B_ (quinone) in a bacterial RC.

The basic experiment consists in making a difference between two IR spectra recorded before and after a given reaction (see Figure 1). All the vibrational bands belonging to molecular groups involved in the reaction will appear in the difference spectrum, whereas all the other vibrations will cancel out without giving any signal. For most reactions, the IR changes induced by the reaction are so small that it is necessary to record the two spectra on the very same sample in the same orientation; in other words, the reaction must be induced inside the sample compartment.

The information in the obtained IR difference spectrum is at an atomic level, so that events like protonation/deprotonation of the side chain of a single amino acid, oxidation/reduction of a pigment or a cofactor, displacement of a water molecule, a protein conformational change, *etc.* can be monitored. In addition, the information gained is also structural: for instance, it is not only possible to observe the formation of a hydrogen bond, but a qualitative assessment on the strength of the bond can also be derived. Finally, time-resolved IR difference spectra can also be recorded. Whereas the “static” IR-DS approach consists of one simple difference spectrum between two states in a biological system, in time-resolved IR-DS [1,2], a series of difference spectra at different times after the beginning of the reaction can be obtained. The reaction can therefore be followed in real time and this dramatically increases the power of the technique: for instance, it allows the concomitance (or non-concomitance) of different events to be established, or transient species to be observed (getting also some information on their molecular structure).

IR-DS is therefore complementary to other biophysical techniques, which give information on specific molecular species. This complementarity is particularly evident in the case of Resonance Raman spectroscopy, where a vibrational spectrum of a chromophore inside a complex biological system can selectively be obtained. Whereas the simultaneous use of several techniques (each one bringing a peculiar piece of information) is particularly useful in the understanding of the mechanism of a biochemical reaction, in the case of IR-DS and Resonance Raman, the synergy is even stronger, as their combined use strongly helps band assignment (especially in the crowded IR-DS spectra, see below).

The huge possibilities of IR-DS are fully exploited when the structure of the system is known (e.g., for proteins, the crystallographic structure) and/or when at least some of the bands appearing in the difference spectrum (or spectra) have been assigned to a vibration of a given chemical moiety (for instance, in proteins, the side chain of a specific amino acid, or a molecular group of a cofactor). In fact, one of the most difficult steps for the analysis of IR difference spectra is called “from bands to bonds”: a series of strategies have been developed to this purpose, including H/D and H_2_^16^O/ H_2_^18^O exchange, site-directed mutagenesis, use of isotopically-labeled pigments and cofactors [1,2]. Theoretical calculations (notably QM/MM and DFT) as well as (in time-resolved spectra) analysis of the kinetic evolutions of bands can also be very helpful [3]. A further difficulty in the interpretation of IR difference spectra comes from the overlap several spectral components. Specific data analysis techniques may be very helpful to tackle this problem [3].

IR-DS has been largely applied to the investigation of photosynthetic systems (see [4,5,6,7] for recent reviews) given the facility of inducing the reaction by light (laser flash or use of a lamp). However, so far most investigations have concerned photosynthetic Reaction Centers (RCs) rather than light-harvesting complexes (LHCs). This is for two main reasons: (a) in RCs, real photo-induced chemistry is taking place (electron transfer, proton transfer, *etc*.), so that the capability of IR-DS to follow—in the same spectrum—the changes in several molecular moieties is a real asset to clarify the mechanism of reaction; and (b) in most—but not all—cases, photo-induced processes in LHCs are fast or extremely fast, so that peculiar techniques are necessary in order to get a IR difference spectrum. Nevertheless, following the pioneering work of Bartel and co-workers [8], in the last 15 years, an increasing number of articles on isolated LHCs or LHC-containing systems have appeared in the literature, mainly related to the use of advanced time-resolved IR techniques with resolution from µs to fs. The list of studied systems include: RC-LHI supercomplexes from purple bacteria [9], chromatophores from purple bacteria ([8,10,11,12,13,14,15] and refs. therein), LHCII from plants [5,16,17,18,19,20], CP43 and CP47 from plants [5], thylakoids [8,21], Peridinin-Chlorophyll *a*-proteins from *Amphidinium carterae* (A-PCP) [22,23,24,25] and *Heterocapsa Pygmaea* (H-PCP) [26]; to this list, a non-light-harvesting protein playing a crucial role in photoprotection of cyanobacteria, Orange Carotenoid Protein (OCP), should be added [27,28].

It should also be mentioned that IR spectroscopy has also been widely used to investigate the interaction of LHCs with metals ([29,30,31] and refs therein). This topic is, however, outside the domain of this review, which is focused on light-induced IR-DS studies on LHCs.

## 2. Instrumental Methods

### 2.1. Static IR-DS

Most IR-DS studies rely on the use of Fourier Transform (FT) IR spectrometers (we will call these studies FTIR-DS). The use of FTIR spectrometers is nowadays widespread and their working mechanism can be found in nearly any textbook of biophysics or physical chemistry. A concise and interesting description can be found in [32,33] (in ref. [32], as well as in ref. [7], several details on its application to photosynthetic systems are given). The detection system is a crucial point: in several FTIR-DS studies, the light-induced signal is so small that the use of a photoconductive or photovoltaic detector is mandatory.

The simplest application of FTIR-DS is the one where the photo-induced process is triggered by a flash or by an (usually short) illumination period (lamp, LED). In some cases, multiple flash sequences have also been used [13]. Two FTIR spectra are recorded before and after the illumination (the spectrum recorded in the dark is used as “background” spectrum). This approach is useful only in case of long-lived processes (τ > ~5 s) because, without the use of specific software, the recording of the first spectrum after the background starts some hundredth of milliseconds (or even more) after the end of the illumination; furthermore, this recording requires at least some seconds. Several applications of this approach have been reported for isolated LHCs, LHC-containing systems or related proteins [13,18,19,27,28]. A modified version of this approach (the difference is made using two spectra from different samples, a procedure which may be applied when relatively big spectral changes are expected) has also been reported [20,21].

A different approach is to use a continuous illumination source (lasting from some tenths of milliseconds to some tenths of seconds). In this case, after the background recorded in the dark, the second spectrum is recorded during the illumination period. This approach makes it possible to “photo-accumulate”, during this period, a short-lived state even without the use of time-resolved techniques (which suffers from a number of drawbacks, see later). It is useful to record a third spectrum also after the end of the illumination period in order to discriminate between spectral contributions from long-lived states (if any) and spectral contribution from short-lived states. This approach has not been used to study isolated LHCs (in [23], it has been tentatively applied to A-PCP without success), mostly because the lifetime of quickly-decaying states (e.g., triplets) is too short to enable their accumulation under illumination. Nevertheless, this approach has been widely used in chromatophores from purple bacteria ([10,11,12] and refs. therein) where LHCs are possibly giving minor spectral contributions to the IR-DS (major spectral contributions are given by RCs). The usefulness of this approach in future studies of isolated LHCs is essentially related to the lifetime of the photo-induced process under investigation.

### 2.2. Rapid-Scan FTIR-DS

**Figure 2:**
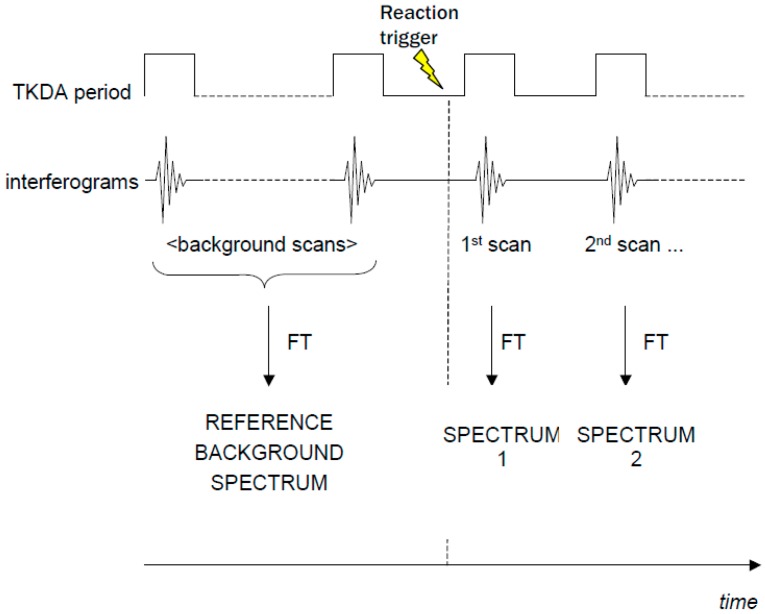
Scheme of a rapid-scan FTIR-DS experiment using a laser flash to trigger the photo-reaction.

Some commercial FTIR spectrometers are equipped with the so-called “rapid-scan” option, which makes it possible to record spectra in very short times (up to ~10 ms). This enables the recording of a series of FTIR spectra after a laser flash or an illumination period in a time-resolved way (see Figure 2). A series of time-resolved FTIR difference spectra can be obtained by subtracting the background spectrum recorded in the dark. This approach has been used to study isolated LHCII from plants [16].

**Figure 3:**
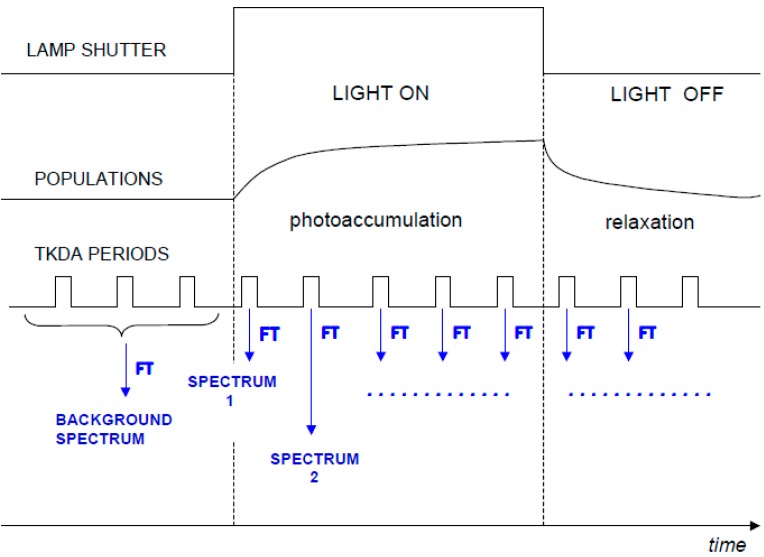
Scheme of a rapid scan FTIR-DS experiment during and after continuous illumination for a hypothetical photoreaction. TKDA periods indicate the time windows during which the interferograms are recorded.

The “rapid-scan” FTIR approach can also be applied in case of continuous illumination. In this case, FTIR spectra are recorded at increasing times after the onset of the light (see Figure 3). Similarly, FTIR spectra can be recorded at increasing time after switching off the light, in order to follow the “relaxation” of the sample (see Figure 3). By subtracting the background recorded before the illumination period, a series of time resolved FTIR difference spectra (during and after illumination) can be obtained. To our knowledge, no studies on isolated LHCs have been reported to date. However, the technique has been successfully applied to chromatophores of *Rb. sphaeroides* [13,14,15] where LHCs are probably responsible for significant contributions to the IR difference spectra (the spectral contributions identified so far are given by quinones in different redox states and phospholipids).

### 2.3. Step-Scan FTIR-DS

**Figure 4:**
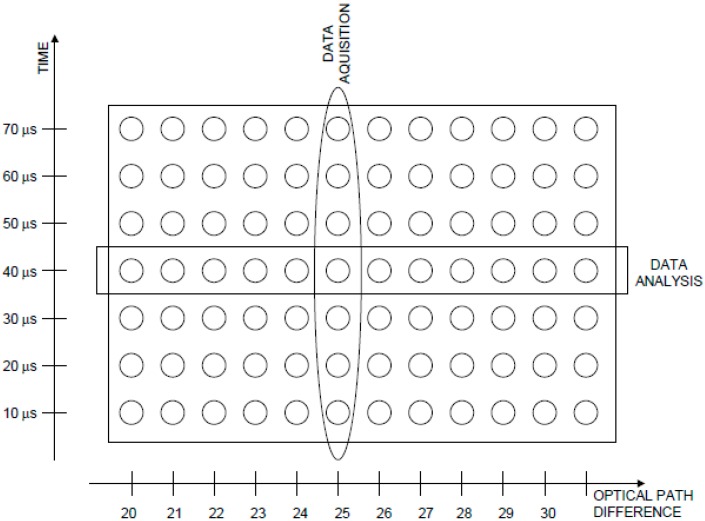
Data array in a time-resolved step-scan FTIR experiment. Data are collected in columns (one column corresponds to each mirror position) and analyzed in rows (each row corresponds to a given time after the flash).

The step-scan FTIR technique is one of the most used in the study of LHCs. It has been applied to A-PCP [22,23,24], H-PCP [26], LHCII [17]. In this technique, the reaction is induced by a laser flash for a series of stop positions of the moving mirror on the Michelson interferometer. Interferograms (and therefore spectra) are reconstructed from a series of experimental data corresponding to a complete series of stop positions of the mirror (see Figure 4 and refs. [1,2,3] for further details).

A clear requirement of the technique is that the reaction must be repeated a large number of times (in practice, at least 10,000, but more often up to several hundred thousands). Some light-induced events in photosynthetic reactions (in LHCs, formation of a triplet state) fulfill this requirement. Nevertheless, it should be kept in mind that, in step-scan FTIR, data acquisition and processing are delicate points [34]; the danger of artifacts is serious and should not be underestimated [35]. In addition, unwanted photothermal effects given by the laser flash triggering the reaction can lead to spectral contributions that can hide (or make less evident) bands originating from the process under study [23,36]. Finally, a careful monitoring of the state of the sample during the experiments (measurement cycles are normally repeated tenths of times, for a global duration of several hours and 10,000–100,000 laser flashes) is strongly recommended. This can be done by (1) checking than no modification in the shape of the differential signals in the IR-DS spectra takes place; and (2) by monitoring—possibly inside the sample compartment of the spectrometer—the UV-Vis-NIR absorption spectrum. Common strategies to minimize sample degradation are to limit laser power (preliminary runs at different laser power are particularly helpful in order to find conditions which maximize the signal and minimize photothermal effects, which are probably related to sample degradation), and to perform experiments at low temperature.

### 2.4. Time-Resolved IR-DS with Monochromator

Flash-induced single-wavelength time-resolved IR-DS has been used to study thylakoids and bacterial chromophores by Bartel *et al.* [8]. Entire time-resolved IR spectra were reconstructed from a series of transients recorded at different wavenumbers. A detailed description of the used instrument can be found in [37].

### 2.5. Ultrafast IR-DS Studies

To record time-resolved IR difference spectra with subnanosecond time resolution, a pump-probe approach has to be employed. Several investigations on LHCs have been carried out, and most of them have been recently reviewed [5]. In the present manuscript, only the ultrafast studies on A-PCP [25] and RC-LHI supercomplexes [9] from purple bacteria—not described in [5]—will be discussed in details, and presented in the more general context of time-resolved IR studies on A-PCP and LHCs from purple bacteria. A detailed description of the experimental method can be found in [5].

### 2.6. Other Techniques

Other techniques for time-resolved IR studies exist, but to date they have not been applied to LHCs. For completeness, we just cite the use of tunable IR lasers and of dispersive IR using an array of detectors, as these two techniques have been successfully used to study photosynthetic RCs ([38,39] and refs. therein).

### 2.7. Data Treatment

Raw IR-DS spectra often need to be elaborated in order to be fully exploitable. Normally, an excellent signal-to-noise ratio is needed, so a common procedure is to average results from several samples. As a rule of thumb, in normal samples the spectral window between 1800 and 1900 cm^−1^ is devoid of spectral contribution and therefore can be used to assess the noise level (which, however, in some spectral ranges like the so-called amide I region—1600–1700 cm^−1^—is usually higher). Other mathematical treatments, like smoothing or baseline correction, are sometimes used but care should be taken not to introduce any artifacts or to distort the spectra.

Data treatment assumes a particularly important role when dealing with time-resolved IR-DS spectra. Indeed, the kinetic behavior of some bands can be particularly helpful to assign them to a peculiar species. It should be taken into account that in IR-DS the overlap of several spectral contributions not only makes the evaluation of the kinetics of single band difficult, but can also lead to unexpected problems, such as band shifts or distortion of band shape. Single wavelength kinetic analysis is particularly convenient for bands that are “isolated” (*i.e.*, they lie in regions free from other spectral contributions) and therefore less prone to the above-mentioned drawbacks. An alternative is the use of chemometric strategies, which can be divided into so-called “soft-modeling” analysis (using approaches that do not rely on any kinetic law for the evolution of the bands) and “hard-modeling” analysis (where a kinetic law—e.g., exponential decays—or even a detailed kinetic scheme is applied to model the time evolution of the spectra). It is beyond the scope of this paper to explore in details the pros and cons of both approaches, but it is important to keep in mind the basic difference between them. Hard-modeling approaches (e.g., target analysis) have been widely used in time-resolved IR spectroscopy of LHCs [5,9,17,22,25,26]. Conversely, soft-modeling approaches, based on Multivariate Curve Resolution (MCR), have just been applied to IR difference spectra from bacterial chromatophores [14]. Mixed “hard–soft” approaches have also been applied [15]. Future possible developments include approaches that have already been successfully used to analyze time-resolved IR difference spectra recorded on isolated RCs, *i.e.*, the use of 2D correlation spectroscopy [40] and the simulation of kinetic profiles using dedicated software [41].

### 2.8. IR Samples

The peculiarity of samples for light-induced IR-DS deserves a dedicated paragraph. The first aspect to be considered is the fact that molar extinction coefficients of vibrational modes are much smaller than those of electronic transitions. This entails the necessity of using very concentrated samples. The normal rule of thumb is to achieve a maximum in absorbance between 0.6 and 0.9 in the amide I region. In addition, given that the bending band of water largely superimposes with the amide I, there is the necessity of getting rid of as much water as possible, provided that the working mechanism of the system under investigation (protein, membrane, *etc.*) is not altered. Alteration of spectral shape and kinetic profiles upon variation of the hydration state of the sample has already been observed in photosynthetic membranes [8,10] and RCs [10,42,43]. The problem should be even more severe for water-soluble proteins. A compromise has therefore to be found between the need of minimizing the water signal and the need of keeping the protein in an environment as similar as possible to the natural conditions.

As already mentioned for step-scan FTIR-DS studies, the intactness and efficiency of the photochemistry/photophysics of the protein should be checked regularly during the repetitions of the IR-DS measurement, by monitoring the shape of IR-DS signals and the UV-Vis-NIR absorption properties of the sample.

Finally, it is important to perform the measurement (which in some cases can last several hours) while keeping the sample under controlled temperature (use of cryostat or of other systems to maintain the sample at constant temperature is strongly recommended).

For more details on the issue of sample preparation, the reader is referred to specific reviews [7,32].

## 3. Bacterial LHCs and Chromatophores

As already mentioned, the first IR-DS investigation on LHC-containing membranes dates back to the mid-1980s, when Bartel and coworkers studied, by time-resolved IR-DS, infrared signals due to photochemical/photophysical processes following a Xenon lamp flash on thylakoids (these results will be discussed later) and chromatophores from three *Rps. capsulatus* mutants (including a mutant devoid of RCs and a mutant devoid of carotenoids) [8]. The authors identified two kinetic components in their time-resolved IR difference spectra following flash excitation in chromatophores where RCs where presents; in the third mutant, devoid of RCs, only one kinetic component was observed [8]. Whereas the slow component—when present—was assigned to photochemistry internal to the RC, the fast component was assigned to energy-dissipating processes [8]. It is interesting to note that the difference spectra associated with this fast component are significantly different in the three mutants; this is most probably related to the different composition of the considered membranes [8]. Despite the recording of time-resolved IR-DS spectra in D_2_O (which entailed interesting band shifts), the authors were not able to provide a molecular explanation for the signals appearing in the difference spectrum of the fast component in the different membranes.

**Figure 5:**
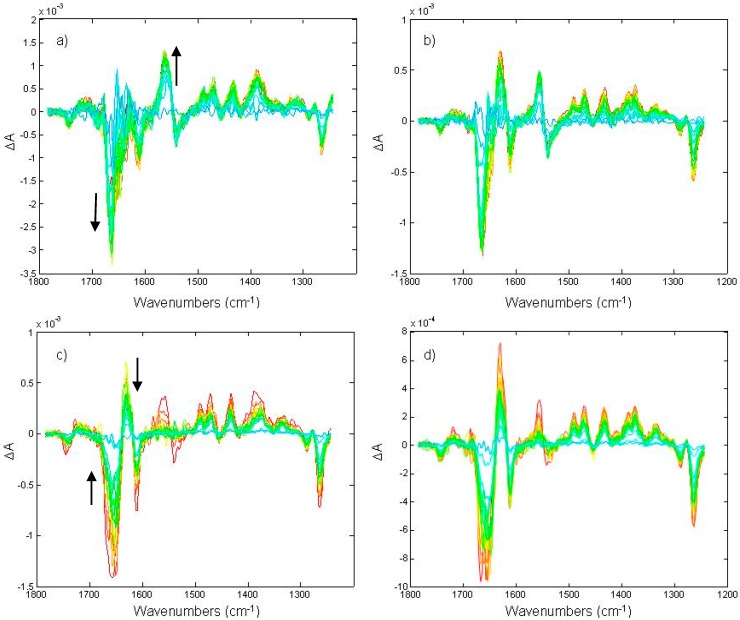
Time-resolved rapid-scan FTIR difference spectra (recorded following the scheme of Figure 3) under (trace **a** and **b**) and after (trace **c** and **d**) continuous illumination at two different light intensities. The duration of the illumination was set to ~4.3 seconds. Positive bands for QH_2_ formation can be observed at 1491, 1471, 1433, and 1375 cm^−1^; negative bands for disappearing Q can be observed at 1288 and 1263 cm^−1^; the differential feature at 1743(−)/1720(+) cm^−1^ was assigned to phospholipid C=O perturbation [13]. The presence of a second kinetic component can be easily deduced by the different kinetics followed by the 1556(+)/1539(−) cm^−1^ differential feature compared to the kinetics of Q and QH_2_ bands. Reprinted with permission from [15], Copyright 2009 American Chemical Society.

Static IR-DS using FTIR spectroscopy was used to study chromatophores of different bacterial species under different conditions by Breton, Nabedryk and co-workers ([11,12] and refs therein) and from Tasumi and co-workers [10]. Despite the fact that conditions were set in order to obtain IR-DS spectra dominated by spectral contributions arising from the RCs, it is likely that minor spectral contributions arise also from other proteins in the membrane, as LHCs. In fact, small but significant discrepancies can be observed when comparing spectra (arising mainly from the same photochemical reaction in the RC) from chromatophores and from isolated RCs. It is interesting to note that the water content in some chromatophores was reported to influence the shape of IR-DS spectra [10]. A more detailed time-resolved IR-DS (using the rapid-scan FTIR technique) under and after continuous illumination at different intensities of chromatophores from *Rb. sphaeroides* was reported by Mezzetti and co-workers [13,14,15] (see Figure 5). Two or three (depending on light intensity) different kinetic components were observed (they could be separated using a soft-modeling MCR approach [14] or a so-called “hard–soft” modeling approach based on MCR [15]). The slow (both under and after illumination) component was found to represent reduction of the quinone pool present in the membrane (positive bands for QH_2_, negative bands for Q). Band assignment for Q and QH_2_ relied on static IR-DS experiments in presence of inhibitors, in D_2_O and using specific flash sequences [13]. The attribution of some other bands, however, remained elusive. The fast (both under and after illumination) component showed mainly spectral contributions arising from the protein (amide I and amide II region) but no clear molecular explanation could be provided [13,14,15]. The third component (present only under strong illumination conditions) also showed—like the slow one—bands characteristic of quinone pool reduction; however, the shape and position of other differential bands was very difficult to interpret. As a working hypothesis, the authors proposed that the two components showing reduction of the quinone pool refer to two different sub-population of the pool itself [15]. The molecular interpretation of the whole fast component, and of several bands in the other two components is still lacking, but it is likely that most—if not all—the observed spectral contributions reflect a reorganization of the membrane after a prolonged illumination, and/or events related to photodissipation of the excess energy. In all three spectral components, signals arising from the RCs are unlikely as in the experimental conditions (multiple quinone reduction per RC) they should be extremely weak.

More recently, Groot and co-workers [9] have used ultrafast IR-DS to investigate isolated RC-LH1 complexes from PufX-plus and PufX-minus *Rb. sphaeroides* membranes. From the analysis of the data, they could conclude that one of the functions of the protein PufX in the photosynthetic membrane is to regulate the photochemistry internal to the RC. Interestingly, a putative spectral contribution arising from excited LH1* was identified at 1635 cm^−1^ and associated with a negative band (arising from ground-state LH1) at 1649 cm^−1^.

To summarize, IR-DS made it possible to investigate: (a) the process of dissipation of excess energy in bacterial chromatophores; (b) the photoreduction of the quinone pool (suggesting the existence of two different sub-pools with different kinetic evolution under illumination); and (c) some possible reorganization of the membrane after a prolonged exposition to light. The obtained information is still very partial, and no detailed interpretation of the observed IR signals (apart from the quinone/quinol bands) could be provided. Further experiments will be required to better understand these processes. Nevertheless, it is important to underline that the observed phenomena could be observed only thanks to the use of IR-DS spectroscopy, which can detect small changes in any constituent of the bacterial photosynthetic membrane.

## 4. Peridinin-Chlorophyll-*a*-Proteins

PCPs are water soluble LHCs from dinoflagellates, which play a key role in light-harvesting and photo-protection [44]. They are also widely used as fluorophores in biomedical research [45]; recently, their coupling with inorganic structures has been reported [44].

**Figure 6:**
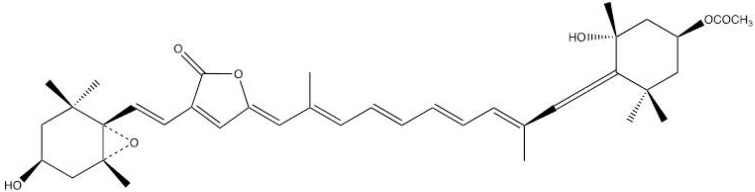
Structure of Peridinin.

PCPs have a peculiar pigment composition (with usually a 4:1 peridinin/chlorophyll ratio; however PCPs with different stoichiometry ratio exist). The PCP protein most studied by IR-DS is A-PCP [22,23,24,25], where 8 Peridinins (Pers) and 2 Chl-*a* are present, arranged in two pseudo-identical domains of four Pers and a Chl *a* molecule. The presence of Per molecules (see Figure 6) in the PCPs enables dinoflagellates to collect light in spectral regions where Chlorophyll poorly absorbs. On the other hand, Per also plays a key photoprotective role as it quenches ^3^Chl *a* (which could be a source of singlet oxygen). The structure of several PCPs has been solved to high resolution [46], and this makes it possible to give a deep interpretation of the spectroscopic data.

Per vibrational properties (in the fundamental and excited states) have been investigated in details by static and time-resolved IR, Raman and Resonance Raman spectroscopy [25,26,47,48,49,50] coupled to DFT [23,24,47,48] and QM/MM [47] calculations; peculiar attention has been paid to the sensitivity of vibrational bands to the surrounding environment [47,48]. The main conclusions are: (1) the stretching mode of the lactonic carbonyl, lying at the typical wavenumber for unsaturated lactones despite its conjugation with the C=C chain, is sensitive the polarity of the environment, not only through the simple band shift effect related to the dielectric constant, but also because of a polarity-modulated Fermi resonance effect between the C=O and an underlying molecular vibration [47,48]; (2) the stretching mode of the lactonic carbonyl is sensitive to the hydrogen bonds that can involve the oxygen atom as an acceptor [47]; (3) the position of the C=C stretching modes and of the allene stretching are almost insensitive to the surrounding environment [47]; and (4) vibrational marker bands for excited Per (triplet state and the so-called Intramolecular Charge Transfer state ICT) have been identified [23,24,25,49,50]. The vibrational properties of Chl *a* have also been studied in details ([26,51] and refs. therein), including (by fluorescence narrowing techniques) in A-PCP [52].

Given the strong overlap of electronic absorption bands due to the different Per in PCPs, ultrafast time-resolved IR-DS represents a unique technique to follow energy transfer mechanism (vibrational bands are very narrow and the position/shape of the C=O stretching band are expected to depend strongly on the local protein environment). As a consequence the spectral contributions from the C=O of the various Pers should be distinguishable. Bonetti *et al.* [25] studied the energy transfer in A-PCP and suggested that large part of the energy transfer from excited Pers to Chl *a* proceeds via S_ICT_ state, localized on two specific Pers, identified as Per 621 and Per 611 (and/or Per 623/613). These two Pers are characterized by a lactonic C=O stretching of 1745 cm^−1^ for the ground state. These assignments have, however, been questioned [44].

Time-resolved IR-DS in the microsecond time domain (by the step-scan FTIR technique) has been widely applied to investigate the process of light-induced triplet formation by two different research groups ([22,23,24,26]; see also [53]). Spectral analysis of IR-DS data recorded at 298 K on A-PCP led van Grondelle and co-workers to the conclusion that two triplet states—with different decay time—are present [22]. The fast-decaying component (τ = 13 µs) was attributed to a triplet state localized on a Per whose lactonic C=O (in the ground state) absorbs at 1745 cm^−1^. The slow component (τ = 42 µs) involves one or two Pers (with lactonic C=O absorbing at 1720 cm^−1^ and 1741 cm^−1^) depending on the excitation wavelength [22]. Most importantly, putative bands reflecting ^3^Chl *a* formation were also identified. According to the authors, this would mean that in A-PCP there is a significant delocalization of the triplet of Chl *a* [22].

**Figure 7:**
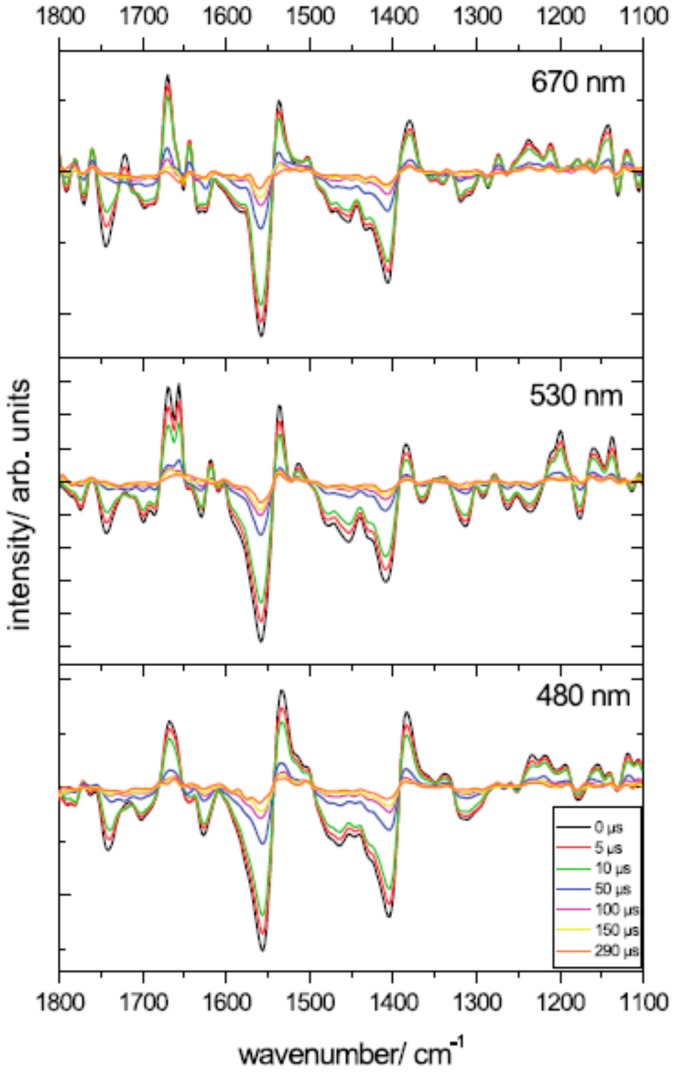
Step-scan FTIR spectra from A-PCP at different times using different excitation laser pulses. T = 298 K. Reproduced from [53].

In addition, as time-resolved IR-DS spectra at 298 K depend on the excitation wavelength used (480, 532, 670 nm; see Figure 7), triplet formation possibly can proceed via different pathways [22]. Step-scan FTIR experiments on A-PCP at 100 K by Mezzetti and co-workers (λ_exc_ = 532 nm) [23,24] confirm the overall shape of the spectra, but indicate that at this temperature probably just one Per (with a lactonic C=O absorbing at 1746 cm^−1^) is involved in triplet formation [23,44]. No clear, unambiguous indication of ^3^Chl *a* involvement was found ([23,24]; see also [44]). Conversely, new putative bands for Per (at 1619, 1357, 1256 cm^−1^) and ^3^Per (1719, 1580, 1534 cm^−1^) were identified in the step-scan FTIR difference spectrum [23,24].

Time-resolved IR-DS spectra of H-PCP at 298 K by van Grondelle and co-workers [26] showed the presence of just one kinetic component (τ = 10 µs), with an associated difference spectrum very similar to the fast-decaying triplet component of A-PCP [22]. Also in this case, presence of putative Chl *a* (negative) and ^3^Chl *a* (positive) bands lead the authors to conclude that—as for A-PCP—Chl *a* is involved in the triplet state. The same research group reported a similar phenomenon—sharing of a triplet between a carotenoid and Chl *a*—also in LHCII from plants (see following paragraph). On this ground, they suggested that triplet sharing represent a molecular mechanism of photoprotection, probably associated with the evolution of oxygenic photosynthesis. It is, however, important to note that advanced EPR results do not suggest the involvement of ^3^Chl *a* neither in A-PCP nor in H-PCP [44].

Several remarks have been made concerning the step-scan FTIR results and the conclusions that were derived [44,48]. For a detailed description, the interested reader is referred to the cited literature. In a few words, the attention was drawn on the fact that (1) experimental step-scan FTIR spectra (especially at 298 K) showed clear evidence of a strong, unwanted phototermal effect [23,44] (see the intense differential band at ~1560(−)/1540(+) cm^−1^ in Figure 7); (2) band assignment for Chl *a* and ^3^Chl *a* in [22,26] are still to be considered as tentative [23,44]; (3) step-scan FTIR is a delicate technique and strong attention should be paid to avoid artifacts and distortions [44]; and (4) both the extracted IR difference spectra and the raw time-resolved IR-DS data at 298 K on A-PCP [53] (see Figure 7) show negative spectral features above 1760 cm^−1^, which suggest that a Fermi resonance effect involving the lactonic C=O of the involved Per(s) may not have been taken into account [48].

As a final remark, it should be noted that, as in the case of the ultrafast IR study, step-scan FTIR-DS made it possible to investigate which, among the different pigments, is bearing the triplet state. This is a consequence of the fact that vibrational bands are very narrow and that some of them (in particular the lactonic C=O of Per) are sensitive to the surrounding environment. The real possibility of disposing of different, well-separated marker bands for each Per in all PCPs is still, however, to be confirmed.

## 5. Orange Carotenoid Protein

**Figure 8:**
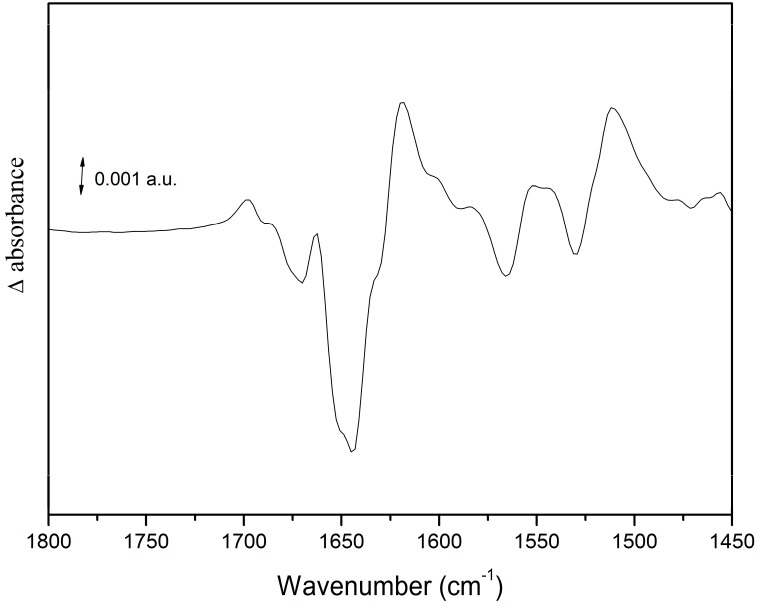
Light-induced FTIR difference spectra on wild-type OCP. See text for further details.

OCP is a photoactive protein that is essential for the photoprotective mechanism of cyanobacteria [54]. Light induces structural changes in OCP from the orange inactive to a red active form, which interacts with the phycobilisome (the cyanobacterial light-harvesting complex). This interaction decreases the energy flowing to the photosynthetic RC. Light-induced IR-DS was applied to study the photo-induced structural changes in isolated OCP [27]. Upon illumination, large spectral changes are observed in the amide I and amide II regions, indicating a strong conformational change. A differential signal at 1677(−)/1697(+) cm^−1^ is assigned to portions of the protein without secondary structure (loops; see Figure 8). An intense and broad negative band is observed at 1643 cm^−1^, whereas positive bands are observed at 1663 and 1618 cm^−1^ (see Figure 8). These signals were interpreted as a less rigid helical structure in a significant part of OCP upon conversion from the orange to the red form, and as a compaction of the β-sheet domain upon photoconversion. The amide I frequency-shift was found to be strikingly similar to the one observed in the LOV2 domain of phototropin (a Flavin-binding blue-light photoreceptor) [55].

IR-DS was then used to investigate the conformational changes of a series of mutants in the framework of a study on the role of N-terminal domain of OCP in phycobilisome binding [28]. All the studied mutants showed a very similar IR-DS spectrum, leading to the conclusion that their response to light is essentially the same of that observed in wild-type OCP.

## 6. LHCII and Other Light-Harvesting Proteins from Plants

LHCII from plants (isolated or membrane-bound) is the LHC that has been studied most extensively by IR-DS. The dynamics of excitation energy transfer within LHCII complexes has been studied by ultrafast IR and summarized in a recent review [5], which also describes the ultrafast IR data obtained on CP43 and CP47, two small antenna proteins present in the Photosystem II core of higher plants [5]. For more details of these ultrafast IR studies, the interested reader is referred to this recent review.

Several studies on isolated LHCII on a longer time-scale have been reported, with the aim of shedding light of the photoprotective role of this protein (see [56] for a recent review). Rogl and co-workers [16] reported that the only spectral changes detected after a light flash lasting 300 µs (Xenon flash tube) using rapid-scan differential FTIR with 60 ms time-scale are those corresponding to light-induced chlorophyll degradation. Gall and coworkers [17] have investigated the light-induced processes after a laser flash at 475 nm by the step-scan FTIR technique coupled to global analysis of the spectra, with a time resolution of ~3 µs. A major spectral component, with a time decay of 20 µs, was identified by the authors, showing putative marker bands for both Chl *a* and lutein. The decay time is typical of carotenoid triplets. These results were interpreted as a sharing of the triplet state between chlorophylls and lutein (similar to those reported on A-PCP and H-PCP, see previous paragraph); such interpretation was strongly corroborated by Resonance Raman spectra at different power, that could provide the C=C stretching band (called ν_1_) for both the ground and triplet state of the carotenoid [17]. A minor spectral component (non-decaying within the time window of the experiment) was also observed and attributed to unquenched chlorophyll (a fraction of Chl molecules that do not transfer the triplet to carotenoids). Looking at the shape of the spectral component and to its long-lived character, we propose instead that this component could correspond to that observed by Rogl and coworkers (and identified as chlorophyll photo-degradation) [16]. It is interesting to note that both the cited works do not report strong protein conformational changes. Finally, it should be mentioned that possibly the step-scan FTIR spectra reported in [17] also show a photothermal effect on the amide II band, as observed for other photoactive proteins [22,36]. Its presence, however, has no direct influence on the proposed interpretation of the step-scan FTIR spectra.

FTIR spectra of monolayer of aggregated of LHCII complexes were recorded under and after a 14-min illumination with blue light by Gruszecki and co-workers [18]. An IR difference spectrum was then calculated, showing an increase of the spectral component in the 1640–1600 cm^−1^ region attributed to a supramolecular organization of the complex, at the expense of the spectral components in the 1690–1660 cm^−1^ region (representing turns and loops structures). This light-driven formation of supramolecular structures is consistent with data from other optical spectroscopy experiments [18].

Interestingly, a very similar “light-minus-dark” IR difference spectrum was obtained by the same research group from IR absorption spectra of lipid protein multibilayers containing LHCII extracted from leaves pre-illuminated with high intensity light (spectrum “light”) and of lipid protein multibilayers containing LHCII extracted from dark-adapted leaves (spectrum “dark”) [20]. It is worth mentioning that in the same paper Atomic Force Microscopy (AFM)-detected IR absorption technique [57] was used to investigate—with an IR imaging approach—the two kinds of lipid-protein multibilayers [20]. The sample containing LHCII from dark-adapted leaves showed an increased signal in the amide I region compared to the other sample; this was interpreted as a sign of the presence of integrated trans-layer structures by vertically aligned LHCII complexes. The interpretation was consistent with AFM analysis (tomography, stiffness) of the samples.

Light-induced IR difference spectra of monolayers of LHCII were recorded at 77 K by Gruszecki and co-workers [19]. The difference spectrum showed, in the 1100–1000 cm^−1^ region, a spectral shift towards higher frequencies of the band representing C-OH stretching vibrations. This shift was interpreted as the result of light-induced process of breaking of hydrogen bonds involving the C-OH group of xantophylls; these results were consistent with data obtained from fluorescence spectroscopy. Possible involvement of photo-isomerization of neoxanthin (a process reported previously by the same research group [58]) was also suggested by the authors.

Time-resolved IR-DS experiments on thylakoids were reported by Bartel and co-workers [8] (in the same paper bacterial chromatophores were also examined, see above). After illumination with a flash from a Xenon lamp (duration 100 µs), two kinetic components, characterized by the same IR difference spectrum, were observed. Their amplitudes were found to depend strongly on the hydration of the sample. The IR difference spectrum showed differential signals arising from water molecules in both the stretching OH region (positive band at 3600 cm^−1^, negative band at 3300 cm^−1^) and in the bending OH region (positive band at 1640 cm^−1^; a weaker negative band is observed at ~1700 cm^−1^ and possibly at ~1550 cm^−1^). The assignment of the 3600(+)/3300(−) cm^−1^ and 1700(−)/1640(+) cm^−1^ differential bands was confirmed by the fact that they were found to shift upon H/D exchange. Some complicated signals are observed between 1150 and 800 cm^−1^ (they were also found to be sensitive to H/D exchange). Experiments under different conditions (different excitation wavelengths, different illumination power, presence of inhibitors, *etc.*) led the authors to the conclusion that these signals represented processes of dissipation of excess energy [8]. The shape of the water differential IR bands strongly suggests that these processes involve water molecules that transiently go from the hydrogen bridged to the free state [8].

A “high light-minus-low light” IR difference spectrum was obtained from IR absorption spectra of thylakoids extracted from leaves of rye illuminated with high intensity light (1200 µmol·m^−2^·s^−1^) and low intensity light (150 µmol·m^−2^·s^−1^) [21]. The difference spectrum showed that in “high-light” thylakoids there is an increase of the 1615 cm^−1^ band (attributed to aggregated LHCII complexes) and a decrease in the 1660–1670 cm^−1^ and 1675–1699 cm^−1^ regions (assigned to turn/loops and to antiparallel β sheets) compared to the low-light thylakoids.

As mentioned at the beginning of the paragraph, LHCII is the light-harvesting protein that has been more extensively studied by IR-DS. Nevertheless, apart from the results obtained by ultrafast IR and step-scan FTIR, a detailed and comprehensive interpretation of all the IR-DS experimental data on LHCII is still difficult. The same can be said for the IR-DS results obtained on thylakoids.

It is, however, clear that IR-DS represent a method of choice, given its capability of monitoring—if necessary in a time-resolved manner—protein conformational changes, protein aggregation and possible changes in the aggregation state of water molecules. Recent papers have also clearly shown how the IR-DS results can be synergically used with results obtained from other techniques (including non-spectroscopic ones).

## 7. Summary and Outlook

IR-DS, both static and time-resolved, has already demonstrated to be an extremely powerful tool to investigate LHCs and LHC-containing systems. The capability of monitoring simultaneously changes on both the pigments (in various electronic states) and in the other constituents of the biological system under investigation is clearly an asset in the study of energy transfer processes and photoprotection mechanisms. The strong increase in the number of published papers on the subject in the last five years clearly suggests that this technique will be used more and more by researchers in the area. Whereas the IR-DS scientific literature on photosynthetic RCs from several organisms is very large (see [4,5,6,7,38,40,42,43] and refs. therein), that on LHCs is still limited. A large number of LHCs are just waiting to be studied by IR-DS.

Several technical advances are likely to contribute to increase the power of the technique. First of all, step-scan FTIR experiments in the ns time scale [34] (to date very limited) are expected to give access to phenomena occurring in a time-scale not much explored so far. Similarly, experiments using dispersive IR instruments [38] are also expected to give a significant contribution, as their sensitivity to absorbance change is increased compared to step-scan FTIR spectrometers [59]. Other experimental approaches (Attenuated Total Reflectance, Surface enhanced Infrared Absorption) [1] will possibly be used in the future.

As already mentioned, classical strategies for band assignment are more difficult to apply compared to RCs (in particular, to our knowledge no IR-DS studies on LHCs with isotopically-labeled pigments have been reported so far). In this framework, the recent development of new approaches for H/D and H_2_^16^O/H_2_^18^O exchange [60], the synergic use of IR-DS, Resonance Raman and fluorescence narrowing techniques [61], advanced calculations to simulate IR spectra of pigments in different states (oxidized, triplet state, singlet excited state, *etc.*) [23,24,47,48], along with parallel vibrational spectroscopy studies of pigments in model environments (e.g., solvents) [47,48], are expected to bring significant contributions. Analysis of so far seldom explored spectral regions (>1800 cm^−1^ and <1000 cm^−1^; their exploration in photosynthetic RCs is becoming more and more popular, see [4,42,62] and refs. therein) and experiments at different hydration levels (as performed in bacterial RCs [42,43]) are also expected to bring new pieces of information, for instance related to the photo-dissipation mechanisms.

Despite the widespread use of established algorithms for the analysis of time-resolved IR-DS, new development in data analysis techniques (for instance, combining classical chemometrical approaches with 2D correlation spectroscopy) will also probably help to interpret the experimental time-resolved IR-DS data.

Recent literature [17,18,19,20,21] has also shown that IR-DS is particularly effective when used in connection with other techniques (fluorescence, AFM, *etc.*), each one bringing a peculiar piece of information, which can be used in a synergic way.

Finally, two research fields that will probably develop in the future are *in vivo* studies and the use of IR Microscopy/nanoscopy. The possibility of *in vivo* investigation is suggested by the large size of some IR-DS signals (e.g., OCP [27,28]); in addition, photo-induced reactions have already been followed by IR-DS at the thylakoid [8,21] or chromatophore level [8,11,12,13,14,15]. The use of IR microscopy/nanoscopy is suggested by a recent investigation on reconstructed photosynthetic membranes [20]. A natural development would also be the combination of these two research approaches aiming to investigate a light-harvesting or a photoprotection mechanism by IR-DS at the level of a single cell/organism or even at a subcellular level, an approach already used for other biochemical reactions [63].
